# Pedagogical models application in physical education on motivational variables, intention to be physically active and basic psychological needs satisfaction: a systematic review with meta-analysis and meta-regression

**DOI:** 10.3389/fpsyg.2026.1897090

**Published:** 2026-07-31

**Authors:** José Manuel Alonso-Vargas, Félix Zurita-Ortega, José Luis Ubago-Jiménez, Pedro Tadeu, Eduardo Melguizo-Ibánez

**Affiliations:** 1 Department of Didactics of Musical, Plastic and Corporal Expression, University of Granada, Granada, Spain; 2 ESECD—CI&DEI—Polytechnic University of Guarda, Guarda, Portugal; 3 Department of Specific Didactics, University of La Laguna, San Cristóbal de La Laguna, Spain

**Keywords:** primary education, secondary education, self-determination theory, student engagement, teaching strategies

## Abstract

In recent years, pedagogical models (PMs) have gained increasing attention in physical education (PE) due to their potential to enhance students' motivational outcomes. This study aimed to systematically synthesize the available evidence on the effects of pedagogical models on autonomous motivation, a motivation, basic psychological needs satisfaction, and intention to be physically active among children and adolescents. A systematic review and meta-analysis were conducted following PRISMA guidelines. Searches were performed in Scopus, Web of Sciences, PsycINFO, SPORTDiscus, ERIC and PsychARTICLES. A total of 20 studies were included in the analysis. Exploratory meta-regression analyses were performed to examine whether content area, pedagogical approach, intervention duration, educational stage, and participants‘ age were associated with the observed effects. The pooled analyses suggested that pedagogical models were associated with higher autonomous motivation (*g* = 0.652; 95% CI [0.373, 0.932]; *p* < 0.001), competence satisfaction (*g* = 0.435; 95% CI [0.252, 0.618]; *p* < 0.001), autonomy satisfaction (*g* = 0.313; 95% CI [0.089, 0.536]; *p* = 0.006), intention to be physically active (*g* = 0.745; 95% CI [0.359, 1.131]; *p* < 0.001) together with lower amotivation (*g* = −0.431; 95% CI [−0.700, −0.162]; *p* = 0.006). Meta-regression analyses indicated that individual sports (β = −1.78; *p* = 0.01) and health-related content (β = −1.68; *p* = 0.02) were associated with lower autonomous motivation compared to team sports. Pedagogical models may promote more favorable motivational outcomes in physical education, particularly autonomous motivation, competence satisfaction, autonomy satisfaction and intention to be physically active. The low certainty of the evidence, substantial between-study heterogeneity and the exploratory nature of the moderator analyses limit confidence in the magnitude of these pooled effects. These findings should be interpreted with caution, and further high-quality randomized controlled trials using more standardized intervention protocols are needed to confirm these associations.

## 1 Introduction

Physical education pedagogy has become an important area of research in recent years (Pill et al., 2024). Traditional teacher-centered approaches have progressively evolved toward student-centered pedagogical models designed to provide more inclusive, meaningful, and positive learning experiences (Backman et al., 2023). Consequently, pedagogical models have emerged as valuable educational tools for addressing contemporary social and educational challenges while promoting students' holistic development (Saiz-González et al., 2024).

Research examining the implementation of pedagogical models in physical education has increased considerably, particularly regarding their effects on students' learning and motivational outcomes (García-Castejón et al., 2021). Pedagogical models are defined as structured instructional approaches that integrate curriculum design, teaching strategies, and learning objectives to facilitate the development of specific competencies (González-Víllora et al., 2019). Although each model has unique instructional characteristics, they share several fundamental pedagogical principles, including active student engagement, meaningful learning, metacognitive development, cooperation, and the promotion of lifelong learning skills (Shen and Shao, 2022). At the same time, each model also presents limitations when implemented in isolation, which has encouraged the development of hybrid approaches (González-Víllora et al., 2019).

Hybridization involves combining the defining characteristics of two or more pedagogical models or integrating complementary elements from different models within the same instructional design (González-Víllora et al., 2019). This approach has gained increasing attention because it allows teachers to capitalize on the strengths of individual models while minimizing their limitations (Joyce, 2014). Previous research has reported positive effects of hybrid pedagogical models across a range of physical education contexts, with improvements observed in students' motor, cognitive, affective, and social development (González-Víllora et al., 2019). Despite the existing evidence on the positive effects of implementing pedagogical models, the literature has indicated that there may be variables that influence their implementation. The type of content to be addressed has been identified as a key element since different approaches involve distinct motor, cognitive, and social demands (Pérez-Pueyo et al., 2021). Similarly, the methodological structure also plays a decisive role. Game-centered models (Teaching Games for Understanding) prioritize tactical understanding and decision-making (Pérez-Pueyo et al., 2021). Models based on sports structure (Sport Education) emphasize affiliation, roles, and the authenticity of the sports experience (Pérez-Pueyo et al., 2021). Social-focused approaches explicitly promote cooperation, responsibility, and interpersonal relationships (Pérez-Pueyo et al., 2021). For their part, innovative models often integrate emerging elements oriented toward creativity, autonomy, and adaptation to changing contexts (Pérez-Pueyo et al., 2021). These methodological differences could have varying effects on the fulfillment of basic psychological needs and, consequently, on students' motivational outcomes. The duration of the intervention has been identified as a key factor in the consolidation of learning and the internalization of motivation (Fernández-Río et al., 2022). The educational stage is a relevant variable, given that evidence suggests differences in motivation levels and perceptions of physical education depending on students' age (Fernandez-Rio and Iglesias, 2022).

Positive experiences in physical education classes contribute to positive attitudes toward physical activity in adulthood (White et al., 2021). These experiences are key factors in the incorporation of physical activity into adult life (White et al., 2021). Motivational processes must be taken into account as they predict engagement in intentional behaviors (Ryan and Deci, 2017). Self-determination theory (Ryan and Deci, 2000, 2017) is one of the most widely used theories in the context of physical education (Fernández-Río et al., 2022). It explains the motivational processes underlying student behavior (Fernández-Río et al., 2022). Furthermore, self-determination theory posits the presence of various social elements that help satisfy basic psychological needs (Ryan and Deci, 2000, 2017). This theory of human behavior makes a clear distinction between need satisfaction and need frustration (Ryan and Deci, 2017; Vansteenkiste and Ryan, 2013; Vansteenkiste et al., 2020). Each person can experience both perceptions within the same context (Burgueño et al., 2023). It has been demonstrated that need satisfaction and frustration can coexist to varying degrees (Burgueño et al., 2023). The satisfaction and frustration of basic psychological needs will be defined by students' backgrounds (Burgueño et al., 2023).

A student who perceives low need fulfillment in physical education classes perceives fewer opportunities than desired, has indifferent relationships with peers, or does not perform tasks as expected (Burgueño et al., 2023; Vansteenkiste et al., 2020). Conversely, a student who experiences need frustration in physical education classes may feel compelled to act according to the teacher's instructions, incompetent, and excluded by peers (Burgueño et al., 2023; Vansteenkiste et al., 2020). Low need satisfaction and the presence of need frustration are considered asymmetrical (Burgueño et al., 2023; Vansteenkiste et al., 2020). Regarding the implementation of pedagogical models, when a specific pedagogical model is introduced for the first time in physical education classes, both students and the teacher report unsatisfactory results (Calderón et al., 2010; Harvey et al., 2020). These results are primarily due to a lack of knowledge and pedagogical understanding of the model (Calderón et al., 2010; Harvey et al., 2020).

A physical education teacher can meet their students' basic psychological needs by employing a motivational teaching style that promotes autonomy, competence, and relatedness. Students feel a sense of autonomy when they perceive their own actions as meaningful (Fernández-Río et al., 2022; Ryan and Deci, 2017). Similarly, students develop a sense of competence when they feel effective or skilled in the activities they perform (Ryan and Deci, 2017). Their sense of relatedness will be fulfilled when they engage in positive communication and feel connected to their peers (Ryan and Deci, 2017).

The application of self-determination theory to physical education reveals different types of motivation depending on the level of self-determination (Fernández-Río et al., 2022). Although the classical paradigm of self-determination theory remains valid (Ryan and Deci, 2000, 2017), it has been superseded by a distinction between autonomous motivation, controlled motivation, and demotivation. According to this new paradigm (Vansteenkiste et al., 2010), autonomous motivation involves the regulation of behavior through experiences of volition, psychological freedom, and reflective self-support (Vansteenkiste et al., 2010). Following the paradigm proposed by Vansteenkiste et al. (2010), controlled motivation involves regulating behavior through experiences of pressure and coercion to think, feel or behave in a certain way (Vansteenkiste et al., 2010). In this case, behavior has a perceived external locus of causality (Vansteenkiste et al., 2010).

The teacher's motivational approach in physical education classes can influence students' attitudes toward physical activity (Fernández-Río et al., 2022; Saiz-González et al., 2024). The main reason for discontinuing active behaviors is a lack of motivation to engage in physical activity (Fernández-Río et al., 2022). One of the main reasons for developing the intention to be physically active is satisfaction with physical education class (Fernández-Río et al., 2022). It has been shown that satisfaction with novelty is associated with positive outcomes in physical education classes, such as enjoyment, autonomous motivation, or the intention to engage in extracurricular physical activity (Fernández-Río et al., 2022; Viciana et al., 2020). To this end, pedagogical models and their hybridizations can serve as effective tools to help foster healthy and active lifestyle habits among students.

Although pedagogical models differ in their instructional characteristics, they are commonly conceptualized within the Models-Based practice framework (Aggerholm and Hordvik, 2024). These approaches share fundamental student-centered pedagogical principles, including active engagement, meaningful learning, autonomous support, collaboration and responsibility development (Aggerholm and Hordvik, 2024). While acknowledging the diversity of their specific instructional strategies, the present review examined these pedagogical models as a broader intervention category to evaluate the overall effectiveness of Model-Based Practice on motivational outcomes in physical education.

Based on the foregoing, this study aims:

O.1. To identify studies of controlled trials and randomized controlled trials that focus on the application of pedagogical models related to autonomous motivation, demotivation, the fulfillment of basic psychological needs, and the intention to be physically active.

O.2. To estimate the effect size of interventions that apply a pedagogical model addressing autonomous motivation, amotivation, basic psychological needs satisfaction and the intention to be physically active.

O.3. To analyze the role of content type, pedagogical approach, duration, and educational stage on the outcomes associated with the implementation of pedagogical models.

## 2 Material and method

### 2.1 Design

This systematic review and meta-analysis was conducted in accordance with the latest update of the PRISMA statement (Page et al., 2021). Additionally, the study's registration code in PROSPERO is CRD420251032027.

### 2.2 Strategy for searching scientific studies

The systematic review and meta-analysis was conducted between December 2025 and May 2026. The following databases were used for the literature search: Scopus, Web of Science, PsycINFO, SPORTDiscus, ERIC and PsychARTICLES. Table 1 presents the search terms used in each database.

**Table 1:** Complete search strategies used in each electronic database.

Database	Search strategy
Web of science	TS=((“Models based practice” OR “Model-based practice” OR “Pedagogical model^*^” OR “Teaching model^*^” OR “Instructional model^*^” OR “Instructional approach^*^” OR “Pedagogical approach^*^”) AND (“Physical education” OR “Physical activity”) AND (Autonom^*^ OR Competenc^*^ OR Relatedness OR “Basic psychological need^*^” OR Motivat^*^ OR “Self-determination”))
Scopus	TITLE-ABS-KEY((“Models based practice” OR “Model-based practice” OR “Pedagogical model^*^” OR “Teaching model^*^” OR “Instructional model^*^” OR “Instructional approach^*^” OR “Pedagogical approach^*^”) AND (“Physical education” OR “Physical activity”) AND (Autonom^*^ OR Competenc^*^ OR Relatedness OR “Basic psychological need^*^” OR Motivat^*^ OR “Self-determination”))
ERIC (EBSCO) PsychARTICLES (EBSCO) SPORT Discus (EBSCO)	TX ((“Models based practice” OR “Model-based practice” OR “Pedagogical model^*^” OR “Teaching model^*^” OR “Instructional model^*^” OR “Instructional approach^*^” OR “Pedagogical approach^*^”) AND (“Physical education” OR “Physical activity” OR Exercise) AND (Autonom^*^ OR Competenc^*^ OR Relatedness OR “Basic psychological need^*^” OR Motivat^*^ OR “Self-determination”))

In addition, inclusion criteria were applied to the literature search. These criteria were established using the PICO framework (Riva et al., 2012). Table 2 presents the inclusion criteria for this study based on the strategy outlined above.

**Table 2:** Inclusion and exclusion criteria.

Database	Inclusion criteria	Exclusion criteria
Population	Elementary and secondary school students aged 6–16 years	Students outside elementary or secondary school or not aged 6–16 years
Intervention	Implementation of a pedagogical model in physical education to promote autonomous motivation, basic psychological needs satisfaction, or intention to be physically active.	Interventions not based on a pedagogical model or not targeting the specified outcomes
Comparison	Randomized controlled trials or controlled trials including experimental and control groups with pre-test and post-test measurements. Control groups did not receive a pedagogical model	Studies without a controlled design, without pre- and post-test data, or without an appropriate control group
Outcomes	Assessment of autonomous motivation, basic psychological needs satisfaction, or intention to be physically active.	Studies not assessing any of these outcomes
Context	School-based physical education settings	Studies conducted outside school settings
Language	Articles published in English or Spanish	Articles published in languages other than English or Spanish
Study characteristics	Use of validated assessment instruments and reporting of sufficient statistical information (mean, standard deviation and sample size for both groups)	Studies not using validated instruments or not reporting sufficient statistical data for effect size calculation

### 2.3 Strategy for selecting research projects

**Figure 1:**
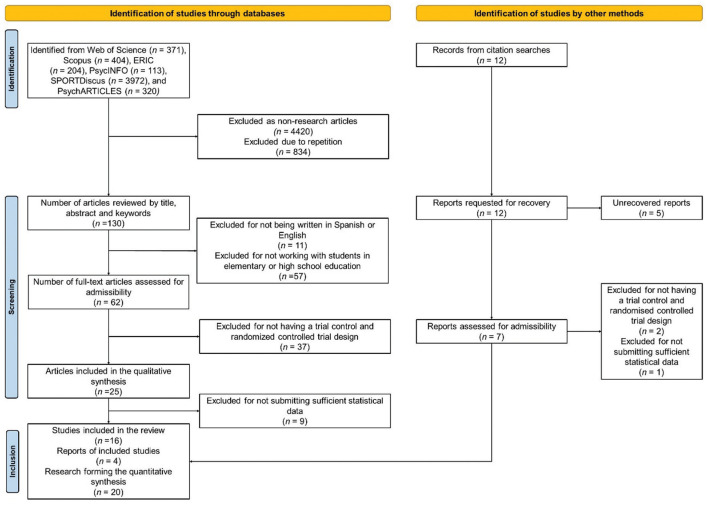
Research flowchart.

Figure 1 presents the research flowchart. A total of 5,384 records were identified through searches of various databases: Web of Science (*n* = 371), Scopus (*n* = 404), ERIC (*n* = 204), PycINFO (*n* = 113), SPORTDiscuss (*n* = 3,972), and PsychARTICLES (*n* = 320). A total of 5,254 studies were excluded for the following reasons: not being research articles (*n* = 4,420) and being duplicates (*n* = 834).

Continuing with the screening phase, 130 scientific articles were reviewed based on their title, abstract, and keywords. Studies were excluded because they were not written in English or Spanish (*n* = 11) or because they did not involve elementary or secondary school students (*n* = 57). We proceeded to examine 62 full-text articles for eligibility. However, 37 studies were excluded due to their design. Twenty-five scientific articles were included in the qualitative synthesis. During the inclusion phase, 9 studies were excluded for not providing sufficient statistical data. As a result of the literature search process, 16 studies were included.

A literature search was also conducted through citation searches. Twelve potential articles were identified. The authors were asked to provide the full-text versions of the studies; however, five studies could not be obtained because no response was received from the authors. As a result, seven studies were evaluated for eligibility. Three studies were excluded for the following reasons: inadequate study design (*n* = 2) and insufficient statistical data (*n* = 1). Based on this, four studies were included through citation searching. Finally, the study sample consisted of 20 studies.

### 2.4 Analysis of the level of bias in the studies

The level of bias was assessed using the Cochrane Bias Tool (Higgins et al., 2011). In this study, bias was analyzed by four authors based on the dimensions outlined in the Cochrane manual (Higgins et al., 2011). This study was conducted to determine whether the studies are influenced by different types of bias. To assess the level of agreement among the authors, Cohen's Kappa coefficient (Cohen, 1998) was used. In this case, the level of agreement among the authors was *K*_*C*_ = 0.854.

### 2.5 Calculation of the effect size

The effect sizes from the various studies were calculated using the Comprehensive Meta-analysis software. To perform these calculations, the research team extracted the mean values, standard deviations, and number of participants from the original studies. Once the aforementioned data were entered, the calculation was performed using sample-size-adjusted standardized mean effects (Sánchez-Meca and Botella-Ausina, 2015). Effect sizes were classified into four levels: null ( ≤ 0.20), small (0.21–0.49), moderate (0.50–0.79), and large (≥ 0.80).

### 2.6 Coding of research studies

The studies included in this study's sample were coded by four researchers. This was done to verify inter-rater agreement in extracting data from the studies and calculating effect sizes. The level of agreement among the researchers was 86.9%. Fleiss's Kappa (*FK*) (Fleiss, 1981) was used to assess the methodological quality of the research. In addition, Cohen's Kappa (*KC*) (Cohen, 1960) was used to assess the quality of the coding. For the first index, a value of *K*_*F*_ = 0.751 was obtained, and for the second, a value of *K*_*C*_ = 0.858 was obtained.

Data were extracted from the studies based on the following criteria (Table 3): (1) Authors (year); (2) Country; (3) Participants; (4) Educational level; (5) Pedagogical model; (6) Content to be covered; (7) Number of sessions. Statistical analysis of meta-analyses and meta-regressions.

**Table 3:** Analysis of the characteristics of the studies included in the quantitative synthesis.

References	Country	Participants	Educational stage	Pedagogical model	Content	Session (duration in minutes)
Méndez-Giménez et al. (2015)	Spain	295 students (14.2 ± 1.68)	Secondary education	Sport education	Ultimate	12 (55 min)
Blatsis et al. (2016)	Greece	215 students (11.61 ± 0.49)	Elementary education	Sport education	Athletics	6 (45 min)
Cuevas et al. (2016)	Spain	86 students (15.65 ± 0.78)	Secondary education	Sport education	Volleyball	19 (55 min)
Menéndez-Santurio and Fernández-Río (2016)	Spain	143 students (15.44 ± 0.80)	Secondary education	Sport education and teaching for personal and social responsibility (hybridization)	Kickboxing	16 (55 min)
Sevil et al. (2016)	Spain	224 students (12.37 ± 0.64)	Secondary education	Tactical games model	Corporal expression	10 (50 min)
Burgueño et al. (2017)	Spain	44 students (16.32 ± 0.57)	Secondary education	Sport education	Basketball	12 (55 min)
Fernandez-Rio et al., 2017	Spain	249 students (13.91 ± 1.76)	Elementary education	Cooperative Learning	Retos Físicos	10 (50 min)
Gil-Arias et al. (2017)	Spain	55 students (15.45 ± 0.41)	Secondary education	Sport education and teaching games for understanding (hybridization)	Volleyball and ultimate frisbee	16 (50 min)
Burgueño et al. (2018)	Spain	44 students (16.32 ± 0.57)	Secondary education	Sport education	Basketball	12 (55 min)
Casado-Robles et al. (2020)	Spain	114 students (14.00 ± 1.1)	Secondary education	Sport education	Deportes alternativos	12 (50 min)
Engels and Freund (2020)	Germany	285 students (12.67 ± 1.10)	Elementary education	Cooperative learning	Cooperative games	16 (50 min)
García-González et al. (2020)	Spain	49 students (15.50 ± 0.57)	Secondary education	Sport education and teaching games for understanding (hybridization)	Volleyball	10 (50 min)
Quintas et al. (2020)	Spain	417 students (11.1 ± 1.7)	Elementary education	Gamification	Dance	12 (55 min)
Segura-Robles et al. (2020)	Spain	64 students (15.00 ± 1.62)	Secondary education	Gamification and flipped classroom (hybridization)	Physical condition and health	8 (50 min)
Viciana et al. (2020)	Spain	123 students (15.50 ± 0.78)	Secondary education	Sport education	Volleyball	12 (55 min)
Franco et al. (2021)	Spain	50 students (14.61 ± 0.50)	Secondary education	Sport education	Basketball	8 (50 min)
García-Castejón et al. (2021)	Spain	99 students (12.63 ± 0.72)	Secondary education	Personal and social responsibility and teaching games for understanding (hybridization)	Futsal, volleyball and basketball	16 (50 min)
Gil-Arias et al. (2021)	Spain	292 students (10.41 ± 0.49)	Elementary education	Sport education and teaching games for understanding (hybridization)	Handball	16 (50 min)
Fernández-Río et al. (2022)	Spain	54 students (14.00 ± 0.10)	Secondary education	Gamification	Fitness and coordination	9 (90 min)
López-Lemus et al. (2023)	Spain	137 students (14.43 ± 0.69)	Secondary education	Sport education and teaching games for understanding (hybridization)	Handball	12 (55 min)

A random-effects meta-analysis was conducted to pool standardized mean differences across studies while accounting for between-study variability. Publication bias was assessed using complementary methods, including Rosenthal's fail-safe *N*, Begg and Mazumdar's rank correlation test, Egger's regression test (Egger et al., 1997) and the Trim-and-Fill procedure. The use of multiple approaches provides a more comprehensive assessment of potential publication bias. Each method evaluates different aspects of funnel plot asymmetry and small-study effects (Fernández-Castilla et al., 2021). Egger regression test exhibits a high degree of specificity, with the effect being (Fernández-Castilla et al., 2021). The test will be statistically significant if *Z* ≥ 1.96 or *Z* ≤ −1.96 (Fernández-Castilla et al., 2021).

The *Q* statistic was used to determine the level of heterogeneity (Sánchez-Meca and Botella-Ausina, 2015). This statistic becomes highly imprecise when few studies are available (Borenstein et al., 2021). Furthermore, this statistic is very conservative; therefore, the significance level was set at *p* < 0.1 (Borenstein et al., 2021). For the reasons cited above, the *I*^2^ statistic was also used to measure the level of heterogeneity. The classification thresholds for heterogeneity were set at 25%, 50%, and 75% to indicate low, moderate, or high levels of heterogeneity, respectively (Borenstein et al., 2021).

Influence diagnostics were performed using studentized residuals and Cook's distance to identify potential outlying or influential studies (Cook, 1977; Viechtbauer and Cheung, 2010). When influential studies were identified, leave-one-out sensitivity analyses were conducted to determine whether their exclusion materially affected the pooled effect estimates or the overall conclusions.

A random-effects meta-regression analysis was conducted to investigate whether heterogeneity among trials could be attributed to the covariates analyzed (Borenstein et al., 2010; Ranganathan et al., 2017). The results of the meta-regressions were interpreted as indicative of an association and of an exploratory nature, due to the moderate number of studies and the observational nature of the comparisons between them (Higgins et al., 2011). Categorical variables were represented using dummy coding, with one category serving as the reference group. This approach allows for estimating the association between each category and the magnitude of the effect size relative to the reference group, while controlling for other predictors (Higgins et al., 2011). Continuous variables provide information on linear associations with effect size, rather than on causal effects (Higgins et al., 2011). A significance level of *p* < 0.05 was adopted.

### 2.7 Certainty of the evidence: GRADE assessment

The certainty of the evidence was assessed using the GRADE framework. The certainty of evidence was rated as low across most outcomes. This downgrade was primarily due to substantial heterogeneity, indicating important variability between studies and to methodological limitations. Some imprecision was detected in several outcomes due to relatively wide confidence intervals. Although risk of bias was generally low to moderate and indirectness was minimal no factors were identified that would warrant upgrading the certainty of evidence. Therefore, the overall confidence in the estimated effects of pedagogical models on motivational and psychological outcomes in physical education is low.

## 3 Results

### 3.1 Analysis of the characteristics of the studies included in the sample

The total sample for the study consisted of 3039 students. In terms of distribution by educational level, 1458 students were in elementary school (47.97%) and 1581 were in secondary school (52.03%). Regarding the distribution of the studies comprising the sample, most of the research was conducted at the secondary education level (*n* = 15; 75.0%). The most used pedagogical model was Sport Education (*n* = 8; 40.0%). In addition, the study also includes studies that combine the Sport Education model with another model (n = 4; 20.0%). Regarding the content to which the pedagogical models are applied, the vast majority focuses on sports instruction (*n* = 14; 70.0%).

### 3.2 Analysis of sample bias

**Figure 2:**
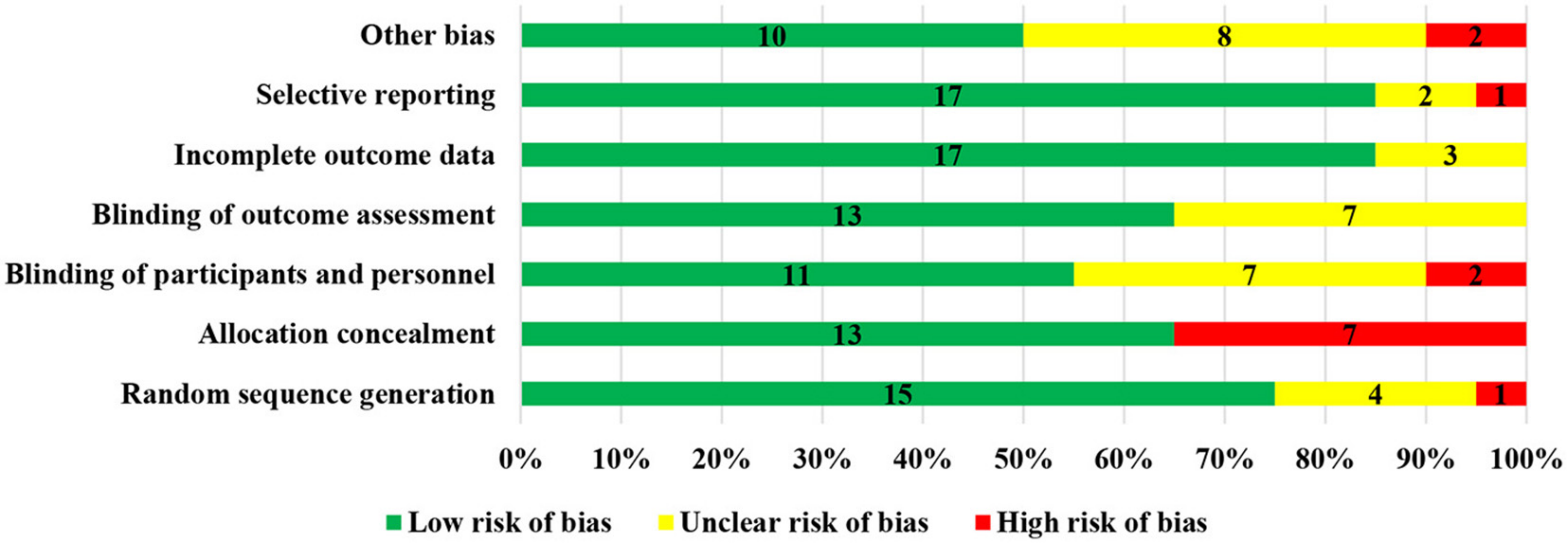
Distribution of bias by analyzed dimensions.

Figure 2 presents the risk of bias assessment for the included studies. Most studies were judged to have a low risk of bias across the assessed domains. Low-risk ratings predominated for random sequence generation, allocation concealment, outcome assessment, incomplete outcome data and selective reporting. The greatest uncertainty was observed for participant blinding and other sources of bias, although only a small number of studies were judged to be at high risk in these domains. The methodological quality of the included studies was considered acceptable.

### 3.3 Publication bias and sensitivity analyses

**Figure 3:**
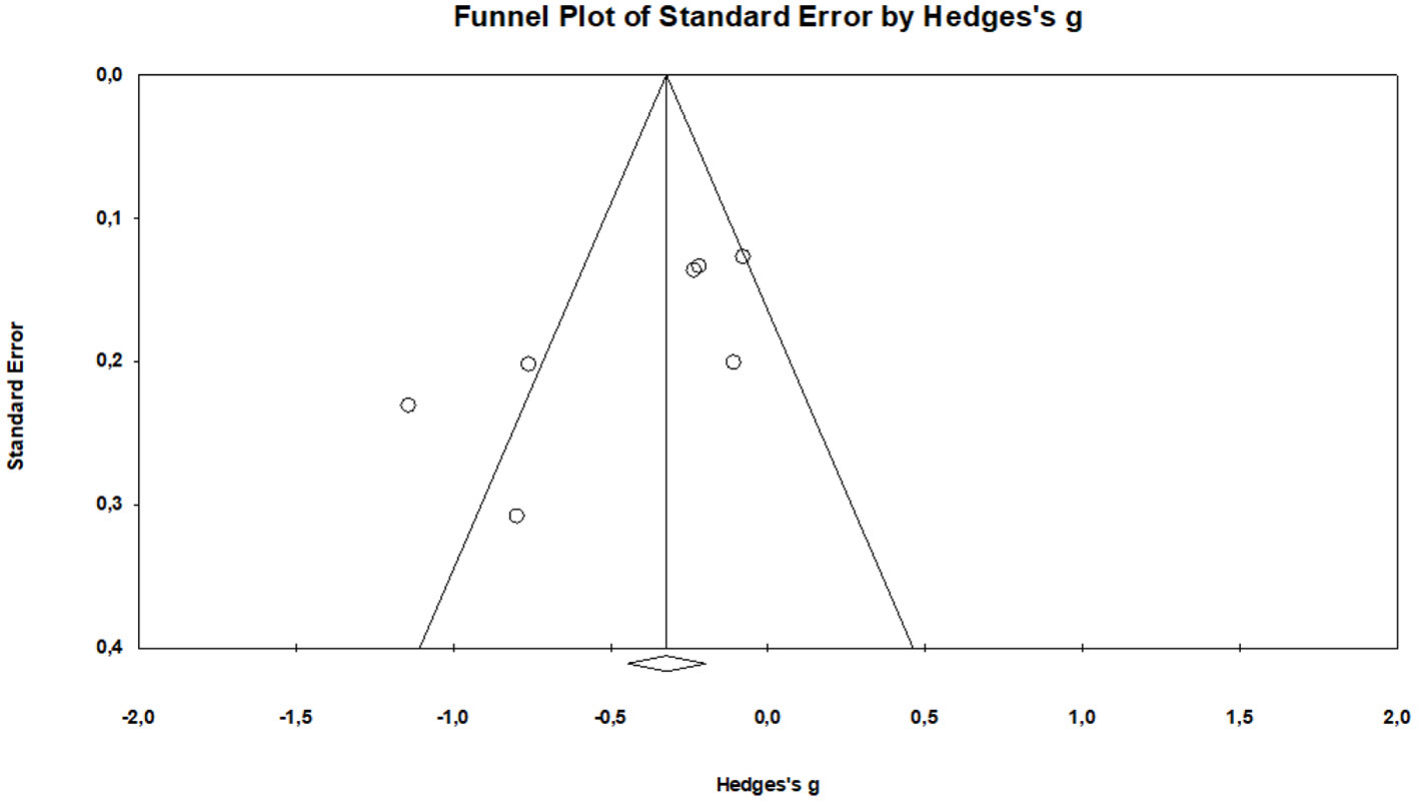
Funnel plot for amotivation.

For autonomous motivation, publication bias was assessed using Rosenthal's fail-safe *N*, Begg and Mazumdar's rank correlation test, Egger's regression test and the Trim-and-Fill method. Rosenthal's fail-safe *N* indicated that 877 additional unpublished studies with null results would be required to reduce the pooled effect to non-significance (Fail-safe *N* = 877, *p* < 0.001), suggesting that the observed effect was relatively robust. Begg and Mazumdar's rank correlation test did not detect significant funnel plot asymmetry (τ = 0.345, *p* = 0.165). In contrast, Egger's regression test indicated significant funnel plot asymmetry (*Z* = 4.725, *p* < 0.001), suggesting the presence of potential small-study effects. Visual inspection of the funnel plot revealed slight asymmetry (Figure 3), although the Trim-and-Fill procedure did not identify any missing studies (0 studies imputed). It indicates that adjustment for potential publication bias did not modify the pooled effect estimate. Influence diagnostics based on Cook's distance did not identify any studies exerting a disproportionate influence on the pooled effect estimate, supporting the stability of the results.

**Figure 4:**
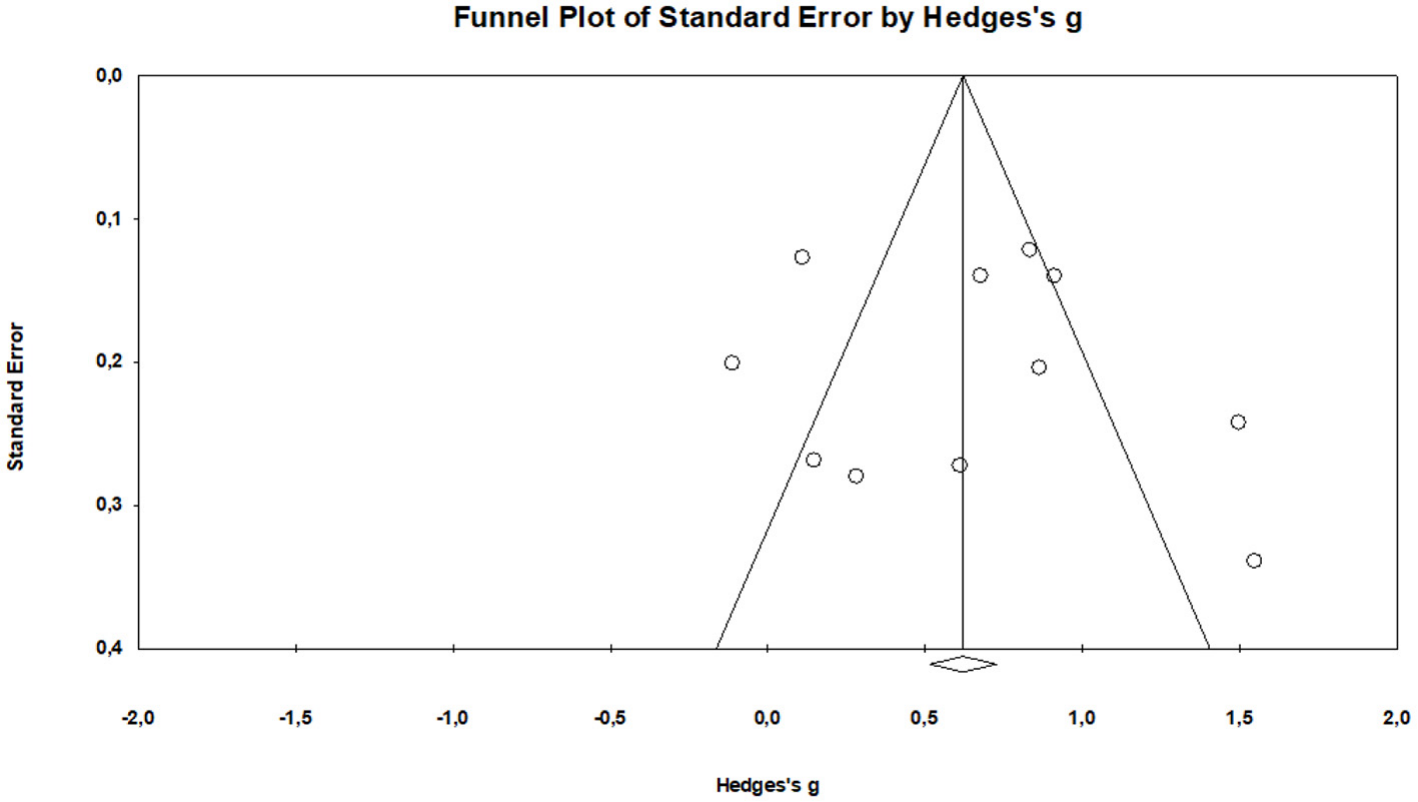
Funnel plot for autonomous motivation.

For amotivation, Rosenthal's fail-safe *N* indicated that 125 additional unpublished studies with null results would be required to reduce the pooled effect to non-significance (Fail-safe *N* = 125, *p* < 0.001), suggesting that the observed effect was relatively robust. Begg and Mazumdar's rank correlation test did not detect statistically significant funnel plot asymmetry (τ = −0.619, *p* = 0.069). In contrast, Egger's regression test indicated significant funnel plot asymmetry (*Z* = −2.309, *p* = 0.021), suggesting the presence of potential small-study effects. Visual inspection of the funnel plot (Figure 4) suggested a slight degree of asymmetry; however, the Trim-and-Fill procedure did not identify any missing studies (0 studies imputed). Influence diagnostics based on Cook's distance did not identify any studies exerting a disproportionate influence on the pooled effect estimate, supporting the robustness of the findings.

**Figure 5:**
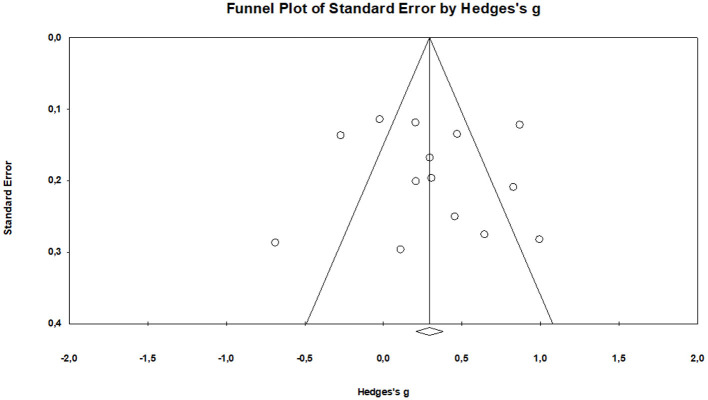
Funnel plot for autonomy satisfaction.

Regarding autonomy satisfaction, Rosenthal's fail-safe *N* indicated that 955 additional unpublished studies with null results would be required to reduce the pooled effect to non-significance (Fail-safe *N* = 955, *p* < 0.001), suggesting that the observed effect was relatively robust. Begg and Mazumdar's rank correlation test indicated significant funnel plot asymmetry (τ = 0.429, *p* = 0.036), which was also supported by Egger's regression test (*Z* = 3.376, *p* < 0.001), suggesting the presence of potential small-study effects. Visual inspection of the funnel plot showed some asymmetry (Figure 5), although the Trim-and-Fill procedure did not identify any missing studies (0 studies imputed). Influence diagnostics based on Cook's distance did not identify any studies exerting a disproportionate influence on the pooled effect estimate.

**Figure 6:**
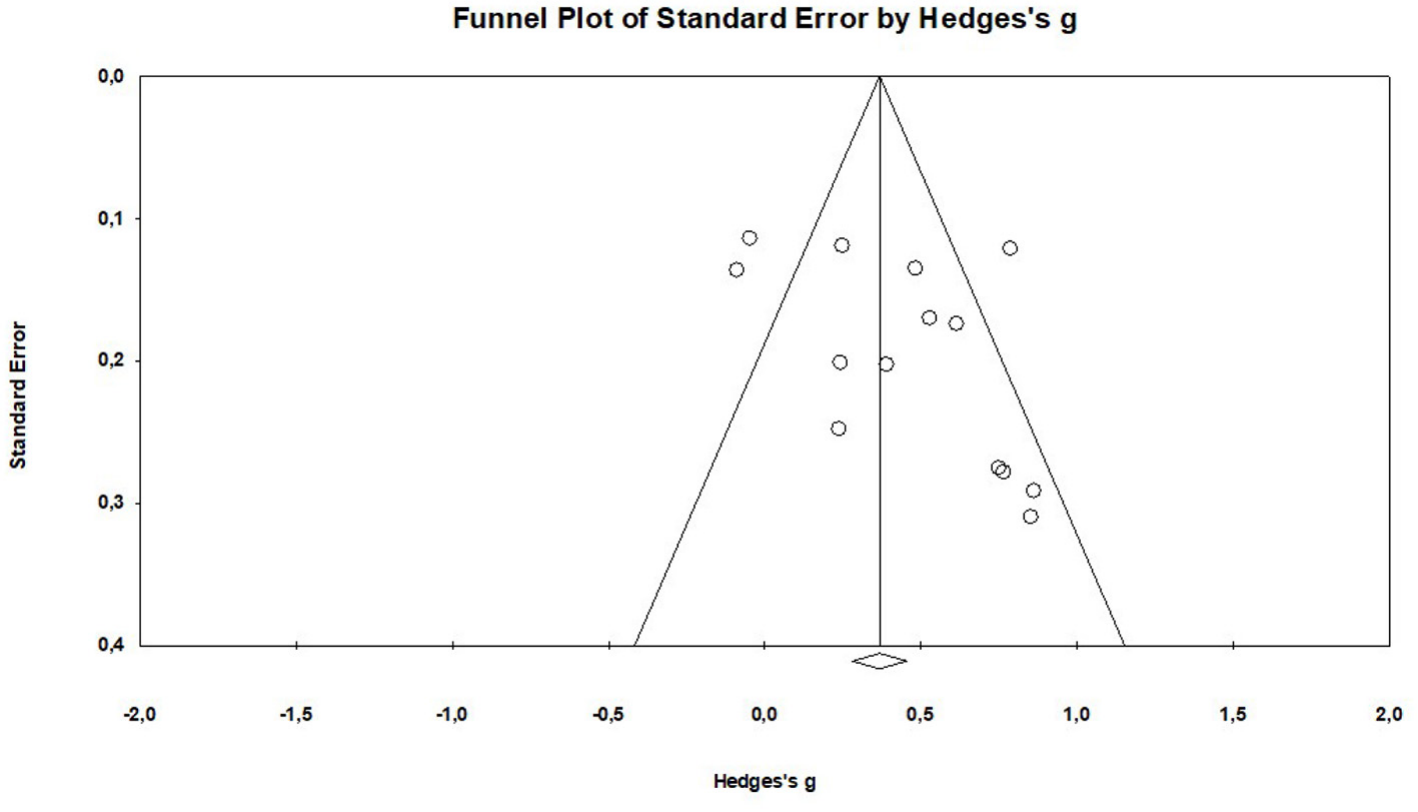
Funnel plot for competence satisfaction.

With respect to competence satisfaction Rosenthal's fail-safe *N* indicated that 550 additional unpublished studies with null results would be required to reduce the pooled effect to non-significance (*p* < 0.001). Both Begg and Mazumdar's rank correlation test (τ = 0.582, *p* = 0.003) and Egger's regression test (*Z* = 3.387, *p* < 0.001) indicated significant funnel plot asymmetry, suggesting the presence of potential small-study effects. Visual inspection of the funnel plot showed some asymmetry (Figure 6), although the Trim-and-Fill procedure did not identify any missing studies (0 studies imputed). Influence diagnostics identified (Segura-Robles et al. 2020) as a potential outlier based on both the studentized residuals and Cook's distance. However, leave-one-out sensitivity analyses showed that excluding this study did not materially alter the overall findings, with the pooled effect remaining moderate and statistically significant (*g* = 0.58, 95% CI [0.30, 0.86]). These results indicate that the conclusions were robust despite the presence of a potentially influential study.

**Figure 7:**
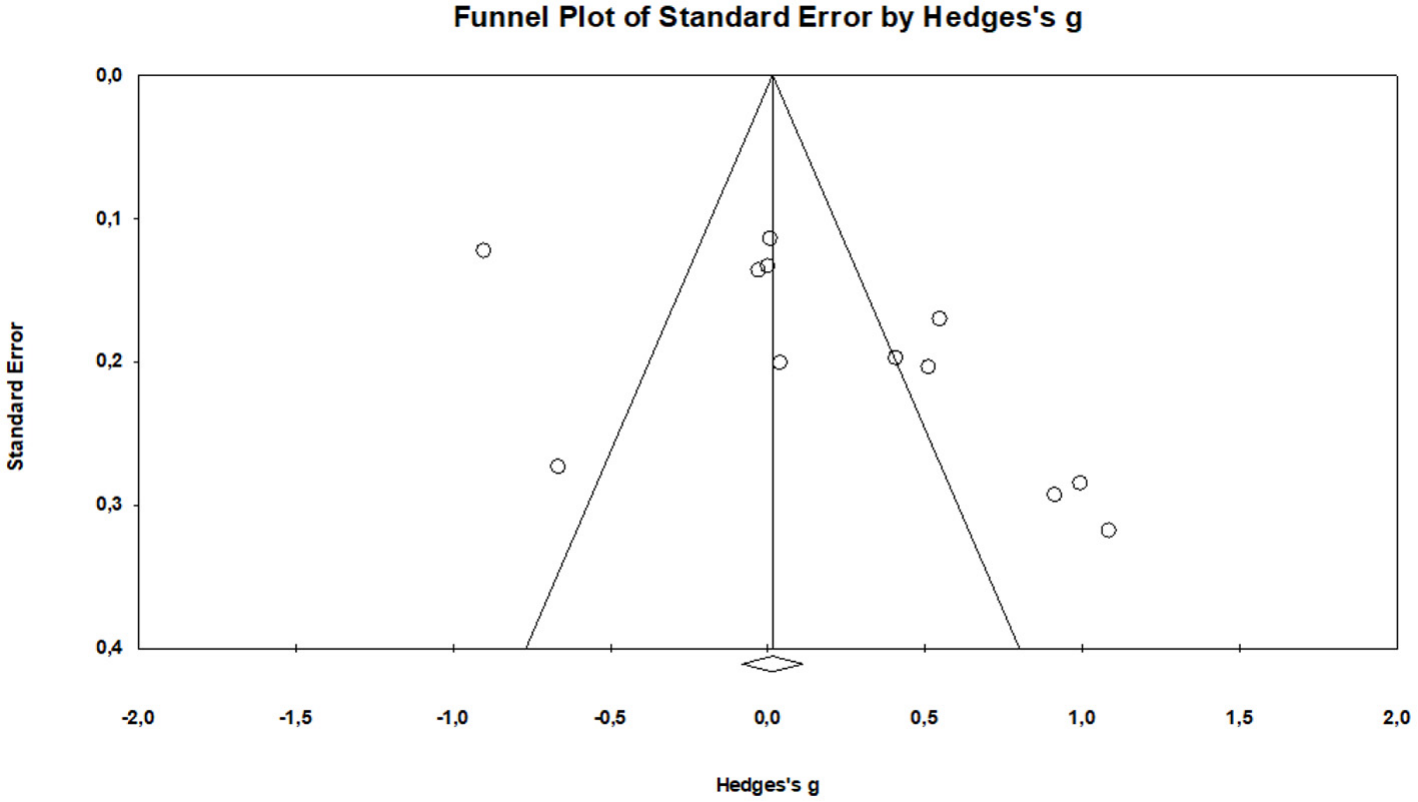
Funnel plot for relatedness satisfaction.

Regarding relatedness satisfaction Rosenthal's fail-safe *N* indicated that 11 additional unpublished studies with null results would be required to reduce the pooled effect to non-significance (*Fail-safe N* = 11, *p* = 0.011). Both Begg and Mazumdar's rank correlation test (τ = 0.455, *p* = 0.045) and Egger's regression test (*Z* = 2.332, *p* = 0.020) indicated significant funnel plot asymmetry, suggesting the presence of potential small-study effects. Visual inspection of the funnel plot was consistent with these findings (Figure 7). The Trim-and-Fill procedure imputed one potentially missing study, indicating that publication bias may have had a limited influence on the pooled effect estimate. Nevertheless, influence diagnostics based on studentized residuals and Cook's distance did not identify any outlying or overly influential studies, supporting the stability of the meta-analytic findings.

**Figure 8:**
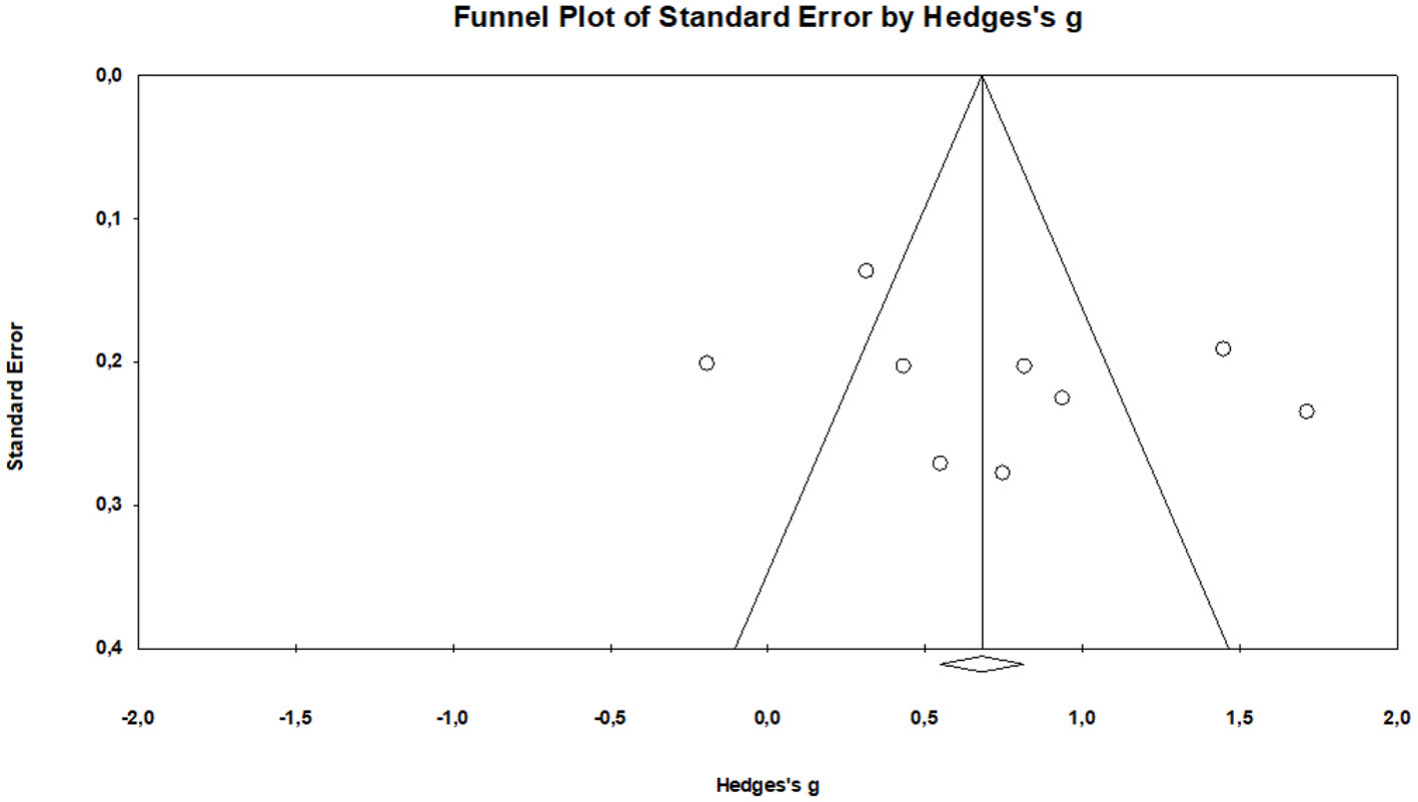
Funnel plot for intention to be physically active.

Finally, for intention to be physically active, Rosenthal's fail-safe *N* indicated that 347 additional unpublished studies with null results would be required to reduce the pooled effect to non-significance (*p* < 0.001), suggesting that the observed effect was relatively robust. Neither Begg and Mazumdar's rank correlation test (τ = 0.222, *p* = 0.477) nor Egger's regression test (*Z* = 0.602, *p* = 0.547) indicated significant funnel plot asymmetry, providing no evidence of potential publication bias or small-study effects. Visual inspection of the funnel plot showed an approximately symmetrical distribution of studies (Figure 8). Consistent with these findings, the Trim-and-Fill procedure did not identify any missing studies (0 studies imputed), indicating that adjustment for potential publication bias did not modify the pooled effect estimate. Furthermore, influence diagnostics based on studentized residuals and Cook's distance did not identify any outlying or overly influential studies, supporting the robustness of the findings.

### 3.4 Analysis of the effect size of the proposals on the variables analyzed

**Figure 9:**
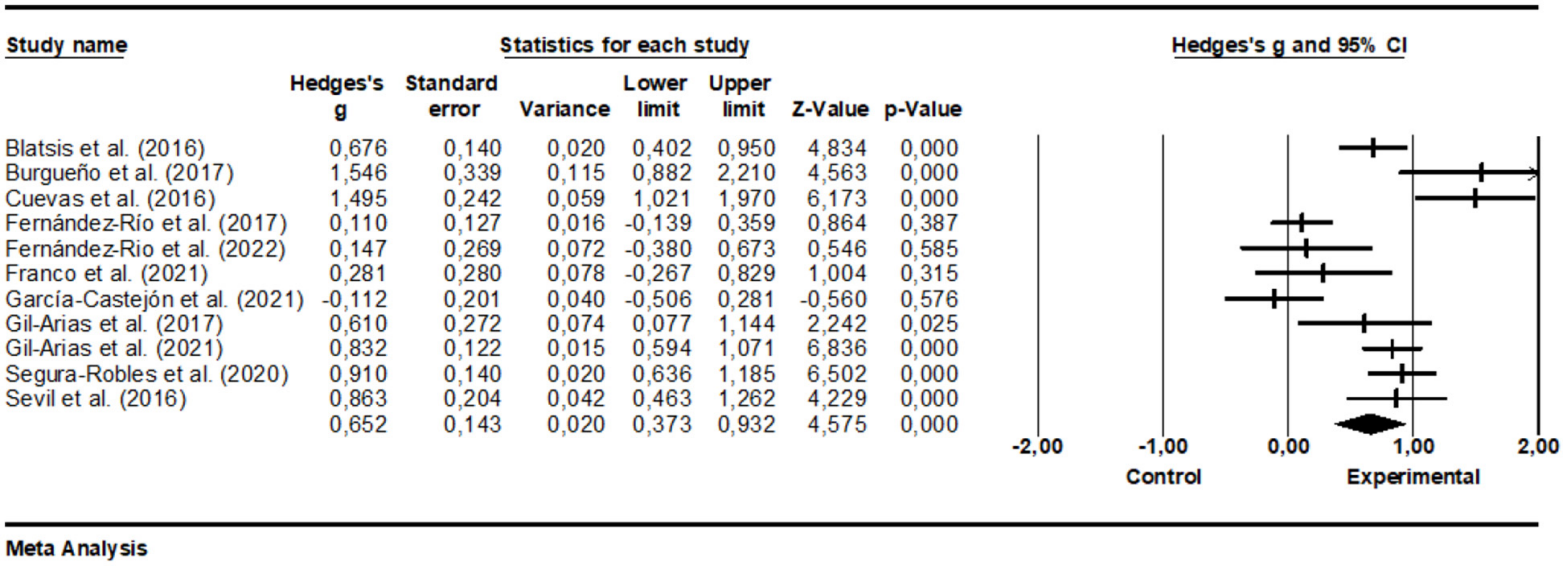
Forest plot of the autonomous motivation variable.

**Figure 10:**
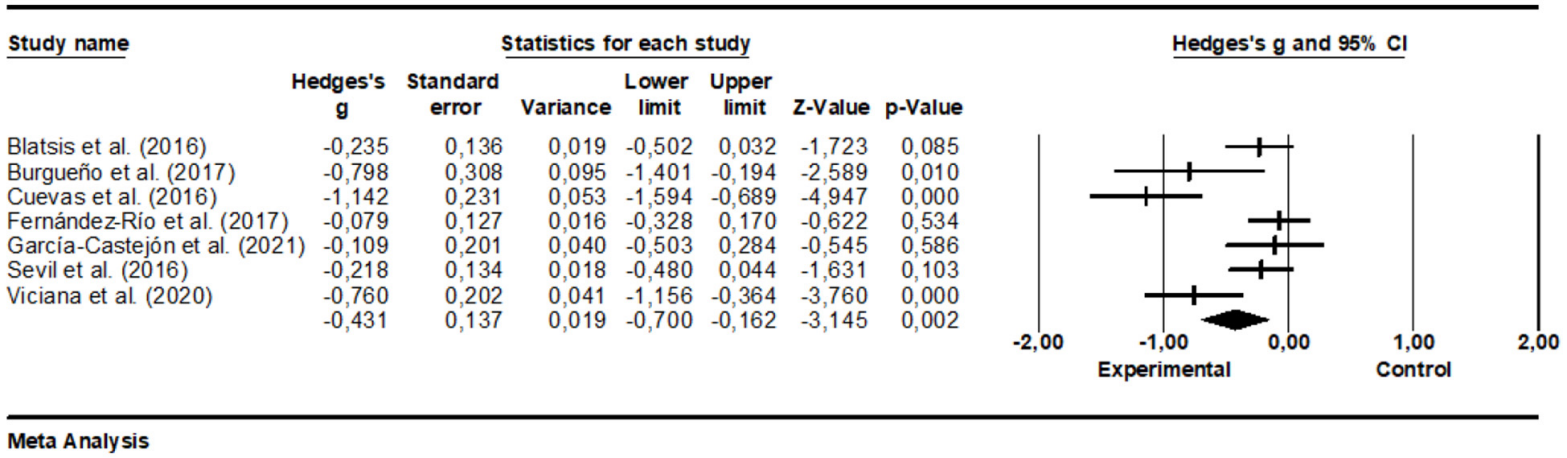
Forest plot of the amotivation variable.

According to the GRADE assessment, the certainty of the evidence ranged from low to very low across all outcomes. The main reasons for downgrading the certainty of the evidence were the substantial between-study heterogeneity, the risk of publication bias and imprecision in some pooled estimates. Although statistically significant effects were observed for several outcomes, confidence in the magnitude and consistency of these effects remains limited. Table 4 presents the effect sizes for the variables analyzed. It shows that the application of pedagogical models focused on autonomous motivation (Figure 9) yields a positive, large and significant effect size (*g* = 0.652; 95% CI [0.373; 0.932]; 95% PI [−0.281; 1.581]; *Z* = 4.575; *p* < 0.001; *Q* = 48.2; *p* < 0.001; *I*^2^ = 75.0%). Similarly, amotivation (Figure 10) has a small but significant negative effect size (*g* = −0.431; 95% CI [−0.700; −0.162]; 95% PI [−1.18; 0.320]; *Z* = −3.145; *p* = 0.002; *Q* = 22.6; *p* < 0.001; *I*^2^ = 72.0%).

**Table 4:** Meta-analysis of the variables examined.

Variable	Participants	Effect size [95% CI]	*I* ^2^	*Z*	*p*
Autonomous motivation	1,855	0.652 [0.373; 0.932]	75%	4.575	< 0.001
Amotivation	1,026	−0.431 [−0.700; −0.162]	72%	−3.145	0.002
Competence satisfaction	2,079	0.435 [0.252; 0.618]	78%	4.663	< 0.001
Autonomy satisfaction	2,048	0.313 [0.089; 0.536]	80%	2.744	0.006
Relatedness satisfaction	1,700	0.212 [−0.113; 0.537]	70%	1.277	0.202
Intention to be physically active	952	0.745 [0.359; 1.131]	74%	3.784	< 0.001

**Figure 11:**
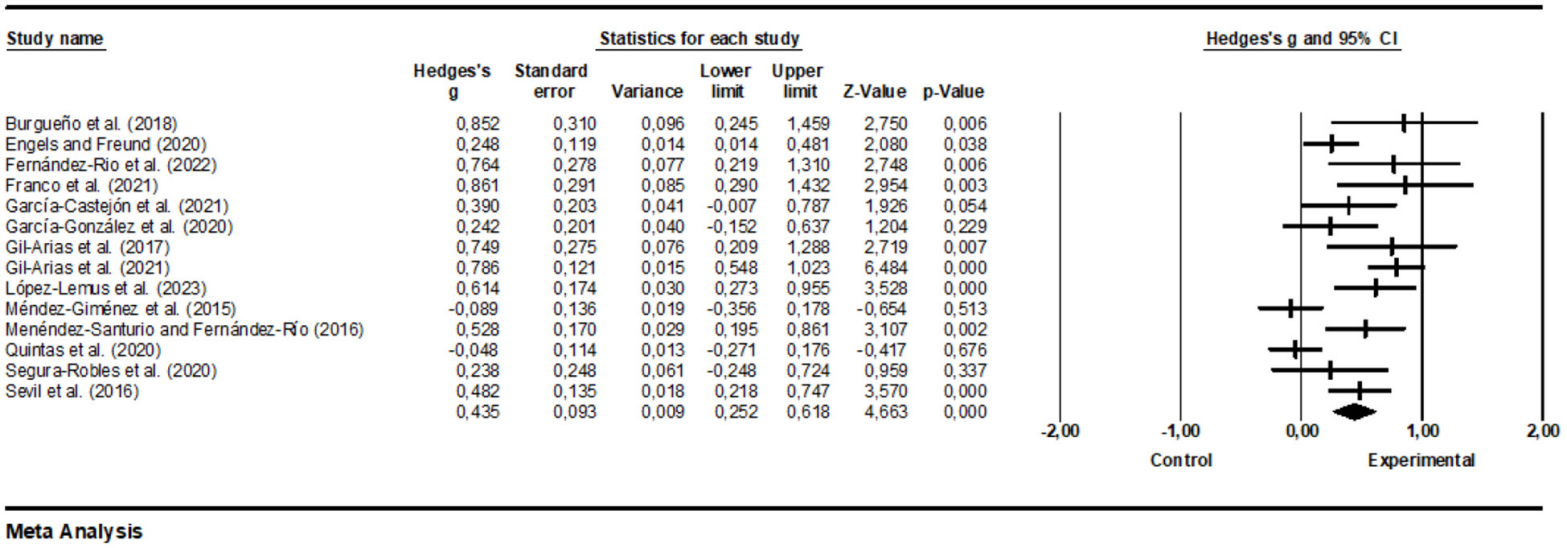
Forest plot of the competence satisfaction variable.

**Figure 12:**
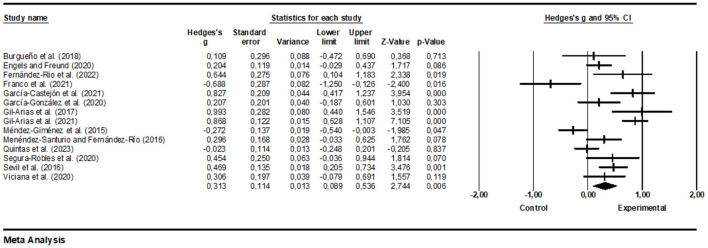
Forest plot of the autonomy satisfaction variable.

**Figure 13:**
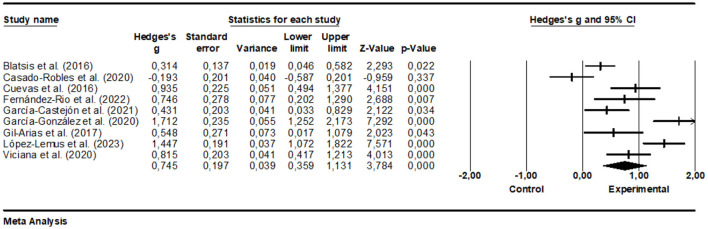
Forest plot of the intention to be physically active variable.

With regard to basic psychological needs, competence satisfaction (Figure 11) shows a moderate and significant effect size (*g* = 0.435; 95% CI [0.252; 0.618]; 95% PI [−0.33; 1.211]; *Z* = 4.663; *p* < 0.001; *Q* = 59.7; *p* < 0.001; *I*^2^ = 78.0%), while autonomy satisfaction (Figure 12) shows a large and significant effect size (*g* = 0.313; 95% CI [0.089; 0.536]; 95% PI [−0.56; 1.181]; *Z* = 2.744; *p* = 0.006; *Q* = 64.9; *p* < 0.001; *I*^2^ = 80.0%). Finally, the use of pedagogical models has a large effect size and significantly influences the intention to be physically active (Figure 13) (*g* = 0.745; 95% CI [0.359; 1.131]; 95% PI [−0.372; 1.863]; *Z* = 3.784; *p* < 0.001; *Q* = 35.8; *p* < 0.001; *I*^2^ = 74.0%).

### 3.5 Analysis of the meta-regression data

Table 5 presents the covariate analyses for the variables examined. An important consideration is the substantial heterogeneity observed across most pooled analyses. Although random-effects models were used to account for between-study variability, these findings indicate that the magnitude of the intervention effects differed considerably across studies. This variability is likely to reflect the diversity of pedagogical models included differences in intervention duration, educational stage, content areas, and variations in study design and implementation. Consequently, the pooled effect sizes should be interpreted as average estimates across heterogeneous educational contexts rather than precise effects that can be expected in all settings.

**Table 5:** Random-effects meta-regression models.

Outcome	Predictor	Reference category	β	SE	95% CI		*Z*	*p*
					Lower	Upper		
Autonomous motivation	Social context	Hybridization	−2.13	1.18	−4.46	−0.46	−1.79	0.07
	Centered games	Hybridization	−1.37	1.20	−3.72	−3.72	−1.15	0.25
	Sports structure	Hybridization	−1.24	0.76	−2.75	−2.75	−1.62	0.10
	Innovators	Hybridization	−0.77	1.22	−3.16	−3.16	−0.63	0.52
	Individual sports	Team sports	−1.78	0.74	−3.25	−0.31	−2.39	0.01
	Body expression	Team sports	−1.63	1.08	−3.75	0.49	−1.50	0.13
	Health	Team sports	−1.68	0.74	−3.15	−0.21	−2.24	0.02
	Number of sessions	–	0.15	0.06	0.02	0.28	2.36	0.01
	Secondary education	Primary education	−1.11	0.57	−2.24	0.009	−1.94	0.05
Amotivation		Individual sports	Team sports	0.46	0.49	−0.50	1.42	0.94	0.34
Body expression		Team sports	0.47	0.49	−0.48	1.43	0.98	0.32
Health		Team sports	0.61	0.48	−0.33	1.57	1.23	0.20
Social context		Hybridization	0.03	0.60	−1.15	1.21	0.05	0.95
Centered games		Hybridization	−0.10	0.60	−1.29	1.07	−0.18	0.85
Sports structure		Hybridization	−0.60	0.49	−1.57	0.36	−1.22	0.22
Number of sessions		–	−0.05	0.03	−0.11	0.009	−1.66	0.09
Secondary education	Primary education	0.41	0.28	−0.14	0.97	1.45	0.14
Competence satisfaction	Social context	Hybridization	−0.32	0.53	−1.36	0.72	−0.60	0.54
	Centered games	Hybridization	0.08	0.42	−0.74	0.90	0.19	0.84
	Sports structure	Hybridization	0.30	0.59	−0.86	1.47	0.51	0.61
	Innovators	Hybridization	0.20	0.59	−0.95	1.36	0.35	0.72
	Number of sessions	–	−0.05	0.04	−0.14	0.02	−1.35	0.17
	Individual sports	Team sports	0.03	0.43	−0.81	0.89	0.09	0.92
	Body expression	Team sports	−0.27	0.31	−0.88	0.33	−0.89	0.37
	Health	Team sports	1.05	0.36	0.32	0.32	2.85	0.0044
	Secondary education	Primary education	−0.35	0.32	−0.99	0.27	−1.10	0.27
Relatedness Satisfaction >cline2-9	Centered games	Hybridization	0.58	0.58	−0.55	1.72	1.01	0.31
Sports structure	Hybridization	0.50	0.50	−0.48	1.48	0.99	0.32
	Innovators	Hybridization	0.55	0.57	−0.57	1.68	0.97	0.377
	Individual sports	Team sports	0.40	0.62	−0.82	1.64	0.65	0.51
	Body expression	Team sports	−0.13	0.46	−1.04	0.77	−0.29	0.77
	Health	Team sports	0.85	0.66	−0.45	2.16	1.28	0.20
	Number of sessions	–	−0.12	0.05	−0.22	−0.02	−2.46	0.01
	Secondary education	Primary education	−0.23	0.63	−1.48	1.02	−0.36	0.71
Autonomy Satisfaction	Social context	Hybridization	−1.32	1.17	−3.63	0.98	−1.13	0.26
	Centered games	Hybridization	−1.06	1.17	−3.37	1.25	−0.90	0.36
	Sports structure	Hybridization	−0.73	0.71	−2.14	0.66	−1.03	0.30
	Innovators	Hybridization	−1.22	0.90	−2.99	0.53	−1.36	0.17
	Individual sports	Team sports	−0.59	0.96	−2.49	1.29	−0.62	0.53
	Body expression	Team sports	−0.67	0.71	−2.07	0.72	−094	0.34
	Health	Team sports	1.54	0.75	0.06	3.06	2.05	0.04
	Number of sessions	–	−0.09	0.08	−0.25	0.05	−1.23	0.21
	Secondary education	Primary education	−0.94	0.57	−2.06	0.16	−1.66	0.09
Intention to be physically active	Sports structure	Hybridization	−0.58	0.38	−1.34	0.18	−1.49	0.13
Innovators	Hybridization	−0.29	0.64	1.55	0.96	−0.46	0.64
Individual sports	Team sports	−0.49	0.67	−1.86	0.83	−0.73	0.46
Health	Team sports	−0.06	0.72	−1.48	1.34	−0.09	0.92
Number of sessions	–	0.0014	0.05	−0.10	0.11	0.02	0.98
Secondary education	Primary education	−0.49	0.63	−1.74	0.75	−0.77	0.44

The exploratory meta-regression analyses provide some insight into potential sources of between-study heterogeneity. For autonomous motivation, significant differences were observed across content areas. Compared with team sports, individual sports were associated with lower autonomous motivation (β = −1.78, *SE* = 0.74, *Z* = −2.39, *p* = 0.01, *95% CI* [−3.25, −0.31]), as was health-related content (β = −1.68, *SE* = 0.74, *Z* = −2.24, *p* = 0.02, *95% CI* [−3.15, −0.21]). In addition, the number of intervention sessions was positively associated with autonomous motivation (β = 0.15, *SE* = 0.06, *Z* = 2.36, *p* = 0.01, *95% CI* [0.02, 0.28]). For competence satisfaction, health-related content (vs. team sports) was positively associated with competence satisfaction (β = 1.05, *SE* = 0.36, *Z* = 2.85, *p* = 0.004, *95% CI* [0.32, 1.32]). Regarding relatedness satisfaction, the number of intervention sessions was negatively associated with this outcome (β = −0.12, *SE* = 0.05, *Z* = −2.46, *p* = 0.01, *95% CI* [−0.22, −0.02]). Likewise, health-related content (team sports) was positively associated with autonomy satisfaction (β = 1.54, *SE* = 0.75, *Z* = 2.05, *p* = 0.04, *95% CI* [0.06, 3.06]). These findings suggest that intervention characteristics, particularly content area and intervention duration, may partially explain differences in the observed effect sizes. However, evidence regarding educational stage was limited and inconsistent across outcomes, and therefore no firm conclusions can be drawn regarding its moderating role.

## 4 Discussion

The findings of this systematic review and meta-analysis suggest that pedagogical models application may improve autonomous motivation, basic psychological need satisfaction and enhance students' intention to be physically active. The GRADE assessment further supports a cautious interpretation of the findings. Although the pooled analyses suggested beneficial effects of pedagogical models on several motivational outcomes, the certainty of the evidence was rated as low or very low across outcomes. Also, substantial heterogeneity was observed across most pooled analyses. Although random-effects models were used to account for between-study variability, the pooled effect sizes should be regarded as average estimates across heterogeneous educational contexts rather than precise effects that can be expected in all settings. The exploratory meta-regressions analyses further suggest that intervention characteristics, particularly session duration and programme length may be associated with differences in the observed effect sizes.

Regarding autonomous motivation and the intention to be physically active, it is observed that the application of pedagogical models has a positive effect on these two variables. Furthermore, these models are found to be effective in reducing levels of demotivation. Previous research points to an increase in motivation and intention to engage in physical activity when pedagogical models are applied (Casado-Robles et al., 2020; Fernández-Río et al., 2022; Viciana et al., 2020). This is mainly due to the promotion of competence when performing any task (Casado-Robles et al., 2020; Fernández-Río et al., 2022; Viciana et al., 2020). A direct and positive causal relationship is observed between increased competence and autonomous motivation (Fernández-Río et al., 2022; Segura-Robles et al., 2020). Furthermore, when a student achieves a high level of competence in performing a task, they feel a sense of fulfillment due to the enjoyment derived from it (Quintas et al., 2020). The opposite occurs when a low level of competence is perceived toward any task (Quintas et al., 2020). When applying any pedagogical model, the goal is for students to foster their autonomy and enjoyment of physical education (García-Castejón et al., 2021). Likewise, it is observed that as autonomous motivation increases, there is a decrease in demotivation toward the task at hand (Méndez-Giménez et al., 2015). About improvements in basic psychological needs, various pedagogical models position the student as a central element in the learning process (González-Víllora et al., 2019). These pedagogical models involve organizing students into small, heterogeneous groups (Fernández-Río et al., 2022). This encourages students to support one another when tackling tasks (Quintas et al., 2020). It also focuses on fostering student autonomy, encouraging them to make decisions that influence the final outcome (Engels and Freund, 2020).

The use of open-ended tasks helps foster students' autonomy (Saiz-González et al., 2024; Fernández-Río et al., 2022). To successfully complete one of these tasks, participants must propose different solutions, fostering critical thinking, creativity, and autonomy (Gil-Arias et al., 2017; Menéndez-Santurio and Fernández-Río, 2016). If the response to this task is successful, students increase their competence in the academic discipline (Segura-Robles et al., 2020). The most

appropriate models for supporting psychological needs are collaborative learning and educational games for comprehension (González-Víllora et al., 2019). Traditional teaching models lead to a decline in students' basic psychological needs (Quintas et al., 2020; Viciana et al., 2020). Traditional teaching styles position the student as a passive element in the teaching-learning process, fostering demotivation toward the physical education subject (Quintas et al., 2020; García-Castejón et al., 2021).

The meta-regression findings should be interpreted as exploratory associations rather than evidence of true moderating effects. Due to the relatively small number of studies available for several analyses and the inclusion of multiple covariates, the statistical power was limited. With regard to autonomous motivation, statistically significant differences were found for individual sports and health-related content compared to team sports. These findings suggest that group contexts may be more effective at facilitating positive social experiences that promote the internalization of motivation (Fernández-Río et al., 2022). Conversely, more individualized contexts may require a more specific pedagogical design to achieve similar levels of motivation (Fernández-Río et al., 2022). Likewise, the number of sessions was positively associated with autonomous motivation, indicating that longer-term interventions promote the development of more self-determined forms of motivation. This result reinforces the need to implement pedagogical models over sufficient periods to consolidate their effects.

Regarding relatedness satisfaction, the only significant predictor was the number of sessions, which showed a negative association. One possible explanation could be that the loss of novelty or the emergence of less positive group dynamics may affect participants' perception of their relationships (Saiz-González et al., 2024). Regarding satisfaction with autonomy, health-related activities have a significant positive effect compared to team sports. This result may be due to the more self-regulated nature of this type of activity, which allows for greater decision-making and adaptation to individual pace (Casado-Robles et al., 2020).

This study is not without limitations that must be considered when interpreting the results. A major limitation is the substantial heterogeneity observed across most analysis. Although random-effects models and exploratory meta-regressions were conducted to investigate potential sources of variability, a substantial proportion of the between-study heterogeneity remained unexplained. The pooled effect sizes should be interpreted with caution. This inconsistency reduces confidence in the precision and generalizability of the pooled estimates and contributed to the low certainty of evidence. Another limitation concerns the meta-regression analyses. Although they provided useful insights into potential sources of between-study variability, these analyses were conducted with a relatively small number of studies in relation to the number of covariates examined. The results may be prone to overfitting and should be interpreted as exploratory associations rather than definitive evidence of moderator effects.

In addition, the current body of evidence presents several methodological limitations. A substantial proportion of the included studies were conducted in Spain. It may limit the generalizability of the findings to other educational systems and cultural contexts. Several studies originated from closely related research groups, potentially reflecting similar methodological approaches and intervention frameworks, thereby reducing the diversity of the available evidence. Another important consideration is that relatively few studies reported detailed information on intervention fidelity, teacher training or implementation quality. These factors are likely to influence intervention effectiveness and may partially explain the variability observed across studies. Although publication bias analyses suggested funnel plot asymmetry for some outcomes, the Trim-and-Fill procedure identified few or no potentially missing studies. Also leave-one-out sensitivity analyses indicated that the pooled effect estimates remained stable following the exclusion of individual studies. Nevertheless, the possibility that studies reporting positive findings are more likely to be published cannot be completely excluded. These limitations reinforce the need to interpret the findings cautiously and highlight the importance of conducting future high-quality randomized controlled trials with improved reporting of intervention implementation and methodological procedures.

Regarding the applicability of the results, this study provides relevant evidence for the educational field, showing that the implementation of pedagogical models can promote motivation, the fulfillment of basic psychological needs, and the intention to be physically active. However, the results also suggest the need to consider variables such as the duration of interventions, the type of content, and the educational stage when designing and applying these models.

In this regard, physical education teachers can benefit from the application of structured, long-term pedagogical models tailored to the characteristics of the student body and the educational context. The combination or hybridization of pedagogical models can be an effective strategy for optimizing motivational outcomes. Finally, these findings can serve as a basis for future research that delves deeper into the analysis of contextual variables, broadens the range of outcomes assessed, and incorporates more homogeneous methodological designs to reduce the observed heterogeneity.

## 5 Conclusions

The findings of this systematic review and meta-analysis suggest that the application of pedagogical models may improve autonomous motivation, promote the satisfaction of basic psychological needs, and enhance students' intention to be physically active. However, these findings should be interpreted with caution because the certainty of the evidence was rated as low according to the GRADE framework.

The exploratory meta-regression analyses suggest that contextual factors such as content type and intervention duration may be associated with differences in the observed effect sizes. However, the evidence supporting educational stage as a potential moderator was limited and inconsistent and should therefore be interpreted cautiously until confirmed by future research. Due to the relatively small number of studies available for several analyses and the number of covariates examined, these findings should be interpreted as exploratory associations. Additional research is required to determine whether these associations are consistent across different educational contexts.

Pedagogical models appear to represent a promising educational approach for enhancing students' motivational outcomes in physical education. However, future well-designed randomized controlled trials with larger samples, more homogeneous intervention characteristics and standardized outcome measures are needed to increase the certainty of the evidence and o confirm the magnitude and consistency of the observed effects.
