# Supplementary material for: Pedagogical models application in physical education on motivational variables, intention to be physically active and basic psychological needs satisfaction: a systematic review with meta-analysis and meta-regression
Source: Front Psychol. 2026 Jul 31;17:1897090. doi: 10.3389/fpsyg.2026.1897090 (PMC13472835; doi:10.3389/fpsyg.2026.1897090)
Supplement: Supplementary file 1 [file Table_1.docx]

Supplementary Material

# 1 Supplementary Figures and Tables

For more information on Supplementary Material and for details on the different file types accepted, please see [here](https://www.frontiersin.org/guidelines/author-guidelines#supplementary-material).

## 1.1 Supplementary Tables

Supplementary Table 1

GRADE analysis

| **Variable** | **Risk of bias** | **Inconsistency** | **Indirectness** | **Imprecision** | **Publication bias** | **Certainty of evidence** |
| --- | --- | --- | --- | --- | --- | --- |
| Autonomous Motivation | Serious | Serious | Not Serious | Serious | Likely | Low |
| Amotivation | Serious | Serious | Not Serious | Serious | Likely | Low |
| Competence Satisfaction | Serious | Serious | Not Serious | Serious | Likely | Low |
| Autonomy Satisfaction | Serious | Serious | Not Serious | Serious | Likely | Low |
| Relatedness Satisfaction | Serious | Serious | Not Serious | Serious | Likely | Very Low |
| Intention to be Physically Active | Serious | Serious | Not Serious | Serious | Likely | Low |
